# Supplementary material for: Content-rich biological network constructed by mining PubMed abstracts
Source: BMC Bioinformatics. 2004 Oct 8;5:147. doi: 10.1186/1471-2105-5-147 (PMC528731; doi:10.1186/1471-2105-5-147)
Supplement: Additional File 2 — The original results of the above study (non-essential files are deleted to keep the file size under the limit set by BMC bioinformatics). [file 1471-2105-5-147-S2.bz2 › chilibotAdditionalFile2/dip05/345ID9261146E4421/html/IL9_IL9R.html]

 


 **IL9** and **IL9R** 
  
Found 39 abstracts in PubMed, retrieved 05.  
 

 What does Google say? 
 PDF only 
| .edu only 

---

**Interactive relationship** (e.g. stimulation, inhibition, etc)

**Non-interactive relationship** (e.g. studied together, co-existance, homology, etc.)

- IL 9  [ **IL9** ]  and IL 9R  [ **IL9R** ]  positive cells were identified with immunocytochemistry, and HCLCA1 expression was detected by means of in situ hybridization with cRNA probes.  Ref: 11842292 J Allergy Clin Immunol, 2002
- CONCLUSIONSIncreased expression of IL 9  [ **IL9** ]  and IL 9R  [ **IL9R** ] , as well as upregulation of hCLCA1, in mucus overproducing epithelium of patients with cystic fibrosis supports the hypothesis that IL 9  [ **IL9** ]  contributes to mucus overproduction in cystic fibrosis.  Ref: 12782818 Laryngoscope, 2003
- Reverse transcriptase polymerase chain reaction of BEAS 2B, HBE4 E6 E7, and primary cells from two subjects revealed messenger RNA for IL 9  [ **IL9** ]  receptor IL 9R  [ **IL9R** ]  alpha but not in BEAS 2B cells.  Ref: 11588013 Am J Respir Cell Mol Biol, 2001
- IL 9  [ **IL9** ] , IL 9R  [ **IL9R** ] , and hCLCA1 expression was determined by immunocytochemical study.RESULTSWe demonstrated significant increases in IL 9  [ **IL9** ] , IL 9R  [ **IL9R** ] , and hCLCA1 immunoreactivity in the mucosa of patients with cystic fibrosis compared with that found in control subjects P < ;.  Ref: 12782818 Laryngoscope, 2003
- We sought to examine the expression of IL 9  [ **IL9** ] , interleukin 9  [ **IL9** ]  receptor IL 9R  [ **IL9R** ] , and hCLCA1 in the upper airway of patients with cystic fibrosis in comparison to healthy control subjects and to demonstrate the relationship of IL 9  [ **IL9** ] , IL 9R  [ **IL9R** ] , and hCLCA1 expression with mucus production.STUDY DESIGNProspective design.METHODSBiopsy samples from nasal polyps of four patients with cystic fibrosis, nasal mucosa of six patients with cystic fibrosis, sinus mucosa of eight patients with cystic fibrosis, and nasal mucosa of six healthy control subjects were stained with periodic acid Schiff PAS to identify mucus glycoconjugates.  Ref: 12782818 Laryngoscope, 2003
- OBJECTIVE We sought to examine the expression of HCLCA1 in bronchial biopsy specimens of asthmatic subjects compared with those of control subjects and to demonstrate its relationship with IL 9  [ **IL9** ] , IL 9  [ **IL9** ]  receptor IL 9R  [ **IL9R** ] , and markers of mucus production.  Ref: 11842292 J Allergy Clin Immunol, 2002
